# Shear Strength of Repaired 3D-Printed and Milled Provisional Materials Using Different Resin Materials with and without Chemical and Mechanical Surface Treatment

**DOI:** 10.3390/polym15214284

**Published:** 2023-10-31

**Authors:** Harisha Dewan, Mohammed E. Sayed, Asayil Jundus, Mafaz Gharawi, Safeyah Baeshen, Maimonah Alali, Mai Almarzouki, Hossam F. Jokhadar, Saad Saleh AlResayes, Mohammed H. D. Al Wadei, Abdulaziz Thubab, Mohammed Jabril Abu Illah, Alkhansa Moafa

**Affiliations:** 1 Department of Prosthetic Dental Sciences, College of Dentistry, Jazan University, Jazan 45142, Saudi Arabia; 2 College of Dentistry, Jazan University, Jazan 45142, Saudi Arabia; 201702596@stu.jazanu.edu.sa (A.J.); 201702622@stu.jazanu.edu.sa (M.G.); 201703429@stu.jazanu.edu.sa (S.B.); 201500320@stu.jazanu.edu.sa (M.A.); 201805583@stu.jazanu.edu.sa (A.T.); 201800026@stu.jazanu.edu.sa (M.J.A.I.); 201808500@stu.jazanu.edu.sa (A.M.); 3 Department of Restorative Dentistry, Faculty of Dentistry, King Abdulaziz University, Jeddah 21589, Saudi Arabia; mzalmarzouki@kau.edu.sa; 4 Department of Oral and Maxillofacial Prosthodontics, Faculty of Dentistry, King Abdulaziz University, Jeddah 21589, Saudi Arabia; hjokhadar@kau.edu.sa; 5 Department of Prosthetic Dental Sciences, College of Dentistry, King Saud University, Riyadh 12372, Saudi Arabia; salresayes@ksu.edu.sa; 6 Department of Restorative Dental Sciences, College of Dentistry, King Khalid University, Abha 61413, Saudi Arabia; moalwadai@kku.edu.sa

**Keywords:** provisional materials, shear strength, 3D printing, milling, resin materials, chemical surface treatment, mechanical surface treatment, restorative dentistry

## Abstract

The aim of this study was to assess the shear bond strength of 3D-printed and milled provisional restorations using various resin materials and surface finishes. There were 160 preliminary samples in all, and they were split into two groups: the milled group and the 3D-printed group. Based on the resin used for repair (composite or polymethylmethacrylate (PMMA)) and the type of surface treatment utilized (chemical or mechanical), each group was further divided into subgroups. The specimens were subjected to thermocycling from 5 °C to 55 °C for up to 5000 thermal cycles with a dwell time of 30 s. The mechanical qualities of the repaired material underwent testing for shear bond strength (SBS). To identify the significant differences between the groups and subgroups, a statistical analysis was carried out. Three-way ANOVA was used to analyze the effects of each independent component (the material and the bonding condition), as well as the interaction between the independent factors on shear bond strength. Tukey multiple post-hoc tests were used to compare the mean results for each material under various bonding circumstances. The shear bond strengths of the various groups and subgroups differed significantly (*p* < 0.05). When compared to the milled group, the 3D-printed group had a much greater mean shear bond strength. When compared to PMMA repair, the composite resin material showed a noticeably greater shear bond strength. In terms of surface treatments, the samples with mechanical and chemical surface treatments had stronger shear bonds than those that had not received any. The results of this study demonstrate the effect of the fabrication method, resin type, and surface treatment on the shear bond strength of restored provisional restorations. Particularly when made using composite material and given surface treatments, 3D-printed provisional restorations showed exceptional mechanical qualities. These results can help dentists choose the best fabrication methods, resin materials, and surface treatments through which to increase the durability and bond strength of temporary prosthesis.

## 1. Introduction

In order to give patients the best oral health and a higher quality of life, restorative dentistry works to restore the shape, function, and appearance of damaged or missing teeth. In order to restore lost or damaged teeth, while the final prosthesis is prepared for installation, provisional prostheses play a crucial part in this process. To maintain patient comfort and correct function throughout the interim period, these provisional prostheses must endure a variety of mechanical pressures, including mastication and occlusal loading [1,2].

Traditional chairside fabrication procedures or indirect techniques utilizing laboratory-based fabrication have been used to create interim restorations in the past [3,4]. The use of computer-aided design and computer-aided manufacturing (CAD/CAM) systems, however, has grown in popularity in dentistry as a result of technological improvements [5]. Using techniques like 3D printing and milling, CAD/CAM systems make it possible to fabricate temporary restorations quickly and effectively while also enhancing accuracy, precision, and reproducibility [6,7].

By permitting the layer-by-layer deposition of materials based on digital models, 3D printing, also known as additive manufacturing, has transformed the field of dentistry. This method has a number of benefits, one of which is the capacity to create specialized provisional restorations with complex anatomical details and exact fits [8,9]. Additionally, 3D printing speeds up the manufacturing of temporary restorations, thereby cutting down on fabrication time—both chairside and in the lab [10]. However, the mechanical characteristics of provisional materials produced via 3D printing may be different from those produced using conventional techniques, which could have an impact on how well they function and last [11].

Milling, which uses subtractive manufacturing with computer-controlled machinery to cut the appropriate shape from a solid block of material, is another extensively used technique for fabricating temporary restorations. Due to the utilization of pre-existing, readily available components in the manufacturing process, milling provides great precision, surface polish, and strength [12,13]. However, milling may produce more waste than 3D printing, and it may not be as effective at creating intricate anatomical designs [12].

For interim restorations to function clinically and last a long period of time, their mechanical qualities are essential. The restoration’s capacity to withstand occlusal stresses and withstand material failure at the adhesive interface is largely determined by shear strength [14]. Provisional restorations are prone to fracture or deterioration during the interim period; hence, repairing them is a routine therapeutic practice [15]. The choice of repair materials and surface treatment techniques, as well as the technique used, all play a role in the restoration procedure’s success [12].

Due to their superior bonding capabilities, the variety of aesthetic possibilities and simplicity of usage, resin materials have become increasingly popular as acceptable repair materials for temporary restorations [16]. To achieve a strong enough connection and long-term durability of the corrected restoration, it is essential to use the right resin substance [17]. Additionally, methods for surface preparation, such as chemical and mechanical processes, have been suggested to strengthen the binding between the repair material and the temporary restoration.

By raising surface energy and encouraging chemical bonding, chemical surface treatments, such as the use of adhesion-promoting chemicals, improve the interaction between the repair material and the temporary restoration surface [18]. Silane coupling agents, which create a chemical link between the resin substance and the substrate, are often utilized adhesion promoters. The temporary restoration surface can be physically altered by sandblasting or roughening to increase the surface area and enhance the mechanical interactions with the repair material [19].

The shear strength of repaired 3D-printed and milled provisional materials utilizing various resin materials with and without chemical and mechanical surface treatments has only been briefly studied, and this is despite the substantial study on provisional restorations and their repair. Thus, the current study’s objective was to assess the shear strength of repaired provisional materials that were 3D-printed and milled using different resin materials, with and without chemical and mechanical surface treatment. The results of this study will aid in the choice of suitable repair materials and surface treatment techniques in restorative dentistry, as well as help to understand the parameters influencing the repair strength of provisional restorations. The hypothesis for the study tests considered whether the shear strength of repaired 3D-printed and milled provisional materials using different resin materials was improved or not after treatment with different chemical and mechanical surface treatments.

## 2. Material and Methods

The in vitro investigation did not require ethical approval. However, the study was initiated after the study protocol was approved by the Institutional Review Board of College of Dentistry, Jazan University (Reference No. CODJU-22061). The material data are summarized in Table 1.

**Figure 1:**
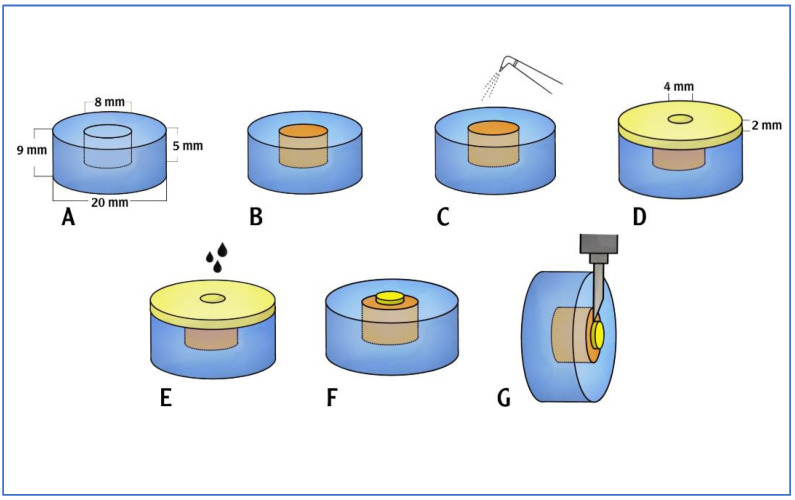
Sample preparation process. (**A**) Sample carrier plastic block with a cylindrical hole. (**B**) The hole to be filled with the temporary crown and bridge material base cylinder. (**C**) Surface treatment was conducted using air abrasion (on the required samples). (**D**) A metal mold with a central cylindrical hole was adapted over the top surface of the sample carrier. (**E**) Surface treatment using etchant and bonding agent was carried out (on the required samples). PMMA/a flowable resin composite material was applied into the mold (on the required samples). (**F**) The prepared sample. (**G**) The shear bond strength testing was carried out.

A total of 160 specimens were fabricated, which were separated into subgroups based on the repair materials (PMMA and resin composite) and manufacturing methods (3D-printed and milled restorations). Figure 1 depicts the sample preparation process. In addition to the 160 specimens, one milled positive control sample and one 3D-printed positive control sample were also fabricated.

### 2.1. Base Cylinder Preparation

**Figure 2:**
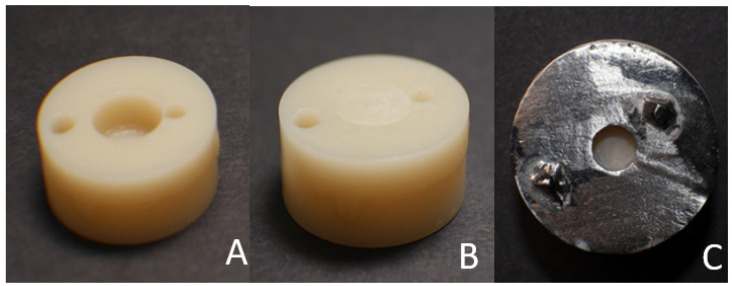
(**A**) Sample carrier plastic block with a cylindrical hole. (**B**) CAD/CAM, either milled or 3D printed, placed in the cylindrical hole. (**C**) A cast-metal mold with a cylindrical hole adapted over the top surface of the sample carrier.

A sample container that measured 20 mm by 9 mm was created utilizing a cylindrical resin block [20]. On one side of the plastic block, a cylindrical hole (8 mm × 5 mm) was made to fit the base cylinder of the test specimens (Figure 2A). Using CAD/CAM, the temporary crown and bridge material base cylinders were created in accordance with the manufacturer’s specifications, which were then inserted into the cylindrical hole of sample container for stabilization upon surface treatment and application of repair materials (Figure 2B). A 5-axis milling machine (DG SHAPE, Roland DGA, Irvine, CA, USA) and PMMA (White Peaks Dental Solutions GmbH, Wesel, Germany) were used to produce 80 CAD/CAM milling base cylinders (8 mm × 5 mm). A corresponding resin material (Asiga, Alexandria, Australia) was used to produce 80 3D-printed base cylinders (8 mm × 5 mm).

### 2.2. Groups Distribution

**Figure 3:**
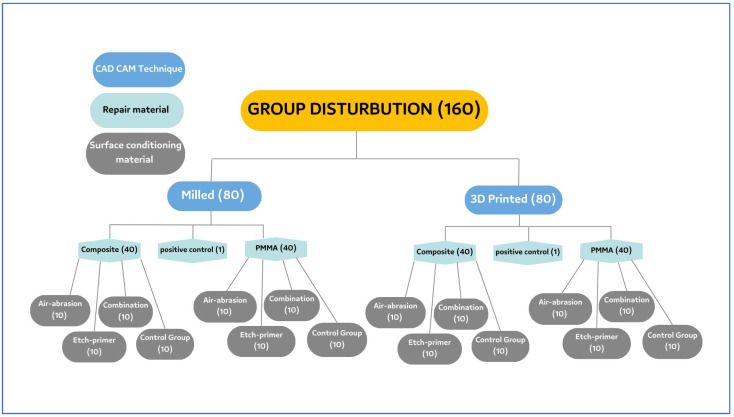
Sample grouping.

Random selection was used to determine which samples of the milled and 3D-printed base cylinders would be subjected to which bonding conditions. The groups are shown in Figure 3.

A total of 80 milled base cylinders were divided into 2 groups (40 cylinders in each group) depending on the type of repair material to be used later on (either composite or PMMA). These groups were further divided into 4 subgroups (*n* = 10) depending on whether they were subjected to no surface treatment, air abrasion, the application of an etchant-primer, or a combination of both the treatments. These treatments were used to condition the surface of the milled base cylinders (Table 2). The groups for milled specimens were as follows:

Group MC (control): Milled base cylinder, no surface treatment, and composite repair material.

Group MCA: Milled base cylinder, air abrasion surface treatment, and composite repair material.

Group MCE: Milled base cylinder, etch-primer surface treatment, and composite repair material.

Group MCC: Milled base cylinder, combination surface treatment, and composite repair material.

Group MP (control): Milled base cylinder, no surface treatment, and PMMA repair material.

Group MPA: Milled base cylinder, air abrasion surface treatment, and PMMA repair material.

Group MPE: Milled base cylinder, etch-primer surface treatment, and PMMA repair material.

Group MCC: Milled base cylinder, combination surface treatment, and PMMA repair material.

A total of 80 3D-printed base cylinders were divided into 2 groups (40 cylinders in each group) depending on the type of repair material to be used later on (either composite or PMMA). These groups were further divided into 4 subgroups (*n* = 10) depending on whether they were subjected to no surface treatment, air abrasion, the application of an etchant-primer, or a combination of both the treatments being used to condition the surface of the 3D-printed base cylinders (Table 3).

The groups for the 3D-printed specimens were as follows:

Group PC (control): A 3D-printed base cylinder, no surface treatment, and composite repair material.

Group PCA: A 3D-printed base cylinder, air abrasion surface treatment, and composite repair material.

Group PCE: A 3D-printed base cylinder, etch-primer surface treatment, and composite repair material.

Group PCC: A 3D-printed base cylinder, combination surface treatment, and composite repair material.

Group PP (control): A 3D-printed base cylinder, no surface treatment, and PMMA repair material.

Group PPA: A 3D-printed base cylinder, air abrasion surface treatment, and PMMA repair material.

Group PPE: A 3D-printed base cylinder, etch-primer surface treatment, and PMMA repair material.

Group PCC: A 3D-printed base cylinder, combination surface treatment, and PMMA repair material.

### 2.3. Sample Completion by Adding Composite/PMMA Repair Material

Repair materials (composite or PMMA) were then added to the specimens in each group after surface treatment.

The preparation of the control group (with composite repair material) applied to the following groups:

#### 2.3.1. Groups MC and PC

**Figure 4:**
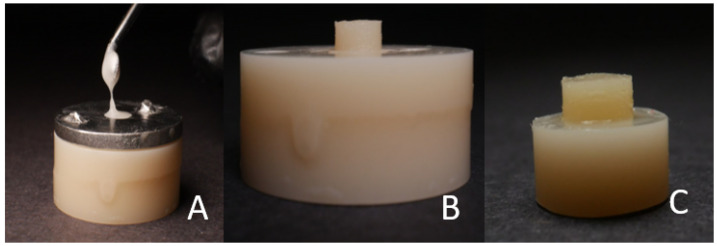
(**A**) The application of the flowable resin composite/PMMA material into the cylindrical hole. (**B**) The sample in the mold. (**C**) The finished sample.

The base cylinders (10 milled and 10 3D-printed) were fitted inside the cylindrical plastic block. A metal delivery mold with a central hole (2 mm thickness and a 4 mm diameter) was fitted on the top surface of the cylindrical plastic block; it was then used to apply the flowable resin composite material to the middle surface of the base cylinders (Figure 2C). The composite resin material (Tetric N-Flow Refill, Ivoclar Vivadent Schaan, Liechtenstein) was dispensed into the cylindrical hole with a tiny overfill. A celluloid strip was placed over the filled cavity and pushed on the plastic block with a glass slide to remove any extra material and to create a level surface. When the material was set, the excess was taken out. The top surface of the mold was covered with a celluloid strip before being pressed with a 1 mm thick glass slide to expel the extra material and to create a level surface. The resin composite was exposed to light for 20 s. The composite resin underwent light curing using an LED curing device for 20 s through a glass slide (Figure 4).

The preparation of the control group specimens (with PMMA repair material) applied to the following groups:

#### 2.3.2. Groups MP and PP

The base cylinders (10 milled and 10 3D-printed) were fitted inside the cylindrical plastic block. The metal mold was fitted on the top surface of the cylindrical plastic block, and it was used to apply the PMMA material to the middle surface of the base cylinders. The test surfaces of the base cylinders (10 milled and 10 3D-printed) were initially wettened with the monomer with a brush. PMMA (UNIFAST III, GC America, Alsip, IL, USA), monomer powder, and liquid were mixed in a dampened dish with a ratio of 1:3 by volume. The mixture was stirred rapidly with a cement spatula for 10–15 s. The acrylic mixture was placed in a single syringe and injected through the hole of the delivery mold (Figure 4A). The acrylic was handled according to the manufacturer’s instructions.

The bottom cylinders were then prepared for the test groups and bonded with different repair materials using 3 different surface treatments.

#### 2.3.3. Groups MCA and PCA

**Figure 5:**
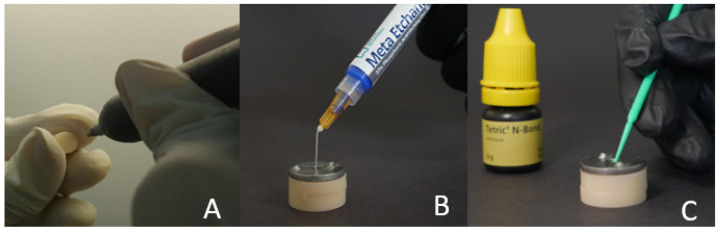
Surface conditioning for the composite using the following methods: (**A**) air abrasion; (**B**,**C**) acid etching-primer application or both.

The test surfaces of the base cylinders (10 milled and 10 3D-printed) were roughened with abrasive grain (110 μm of Al_2_O_3_ particles) (Korex 250, BEGO, Lincoln, RI, USA) at a pressure of 2.5 bar for 5 s (Figure 5A). The base cylinders were then fitted inside the cylindrical plastic block. The metal delivery mold was used to apply the flowable resin composite material to the middle surface of the base cylinders, as described in the control groups MC and PC.

#### 2.3.4. Groups MCE and PCE

The base cylinders (10 milled and 10 3D-printed) were fitted inside the cylindrical plastic block. The metal mold was fitted on the top surface of the cylindrical plastic block. The base cylinder surface was rinsed and dried, and 37% Meta Etchant gel (Meta P and Bond, METABIOMED Co., Ltd., Cheongju, Korea) was applied for 15 s. The gel was then removed and rinsed for 20 s, and it was later blot dried. Two successive coats of adhesive (Tetric N-Bond, Ivoclar Vivadent, Schaan, Liechtenstein) were then applied to the test surface for 15 s, which was then lightly dried with compressed air for 5 s (Figure 5B,C). The adhesive was light-cured for 10 s. The flowable composite resin material was then applied to the middle surface of the base cylinders, as described in the control groups MC and PC.

#### 2.3.5. Groups MCC and PCC

The test surfaces of the base cylinders (10 milled and 10 3D-printed) were roughened with abrasive grain, as in groups MCA and PCA. The base cylinders were then fitted inside the cylindrical plastic block. The metal mold was fitted on the top surface of the cylindrical plastic block. Two successive coats of adhesive were then applied to the test surface after etching, as in groups MCE and PCE. The flowable composite resin material was then applied to the middle surface of the base cylinders, as described in the control groups MC and PC.

#### 2.3.6. Groups MPA and PPA

**Figure 6:**
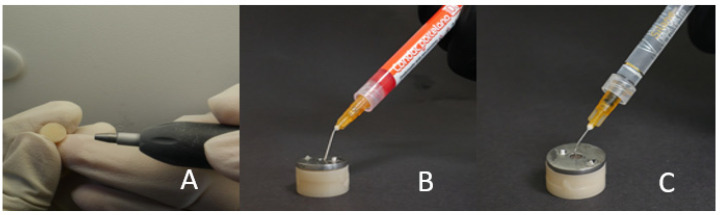
Surface conditioning for PMMA using the following methods: (**A**) air abrasion; (**B**,**C**) acid etching-primer application or both.

The test surfaces of the base cylinders (10 milled and 10 3D-printed) were roughened with abrasive grain, as in groups MCA and PCA (Figure 6A). The base cylinders were then fitted inside the cylindrical plastic block. The metal mold was fitted on the top surface of the cylindrical plastic block, and this was then used to apply the PMMA to the middle surface of the base cylinders, as described in the control groups MP and PP.

#### 2.3.7. Groups MPE and PPE

The base cylinders (10 milled and 10 3D-printed) were fitted inside the cylindrical plastic block. The metal mold was fitted on the top surface of the cylindrical plastic block. Then, 10% hydrofluoric acid (Condac Porcelana, FGM dental Products, Brazil) was applied to the base cylinder through the exposed hole. The etchant was rinsed with abutment water, and this was later dried off. Silane (Ultradent Products Inc., South Jordan, UT, USA) was applied with a mini brush tip (Figure 6B,C). It was left there to evaporate for 60 s, which was then air-dried with oil-free air. The PMMA material was then applied to the middle surface of the base cylinders, as described in the control groups MP and PP.

#### 2.3.8. Groups MPC and PPC

The test surfaces of the base cylinders (10 milled and 10 3D-printed) were roughened with abrasive grain, as in groups MCA and PCA. The base cylinders were then fitted inside the cylindrical plastic block. The metal mold was fitted on the top surface of the cylindrical plastic block. Condac Porcelana 10% and silane were applied to the base cylinder through the exposed hole, as in groups MPE and PPE. The PMMA material was then applied to the middle surface of the base cylinders, as described in the control groups MP and PP.

#### 2.3.9. Positive Control

The combined unit of the base cylinder and the repair material was scanned to generate positive control specimens. Two positive controls were fabricated using the STL file, one entirely milled and the other entirely 3D printed. The positive control was used to provide a reference value for the strength of the non-repaired materials at the studied dimensions for the milled and 3D-printed materials, as well as to develop an idea on how far the repaired specimens differed from the non-repaired positive control specimens.

### 2.4. Shear Bond Strength Test

**Figure 7:**
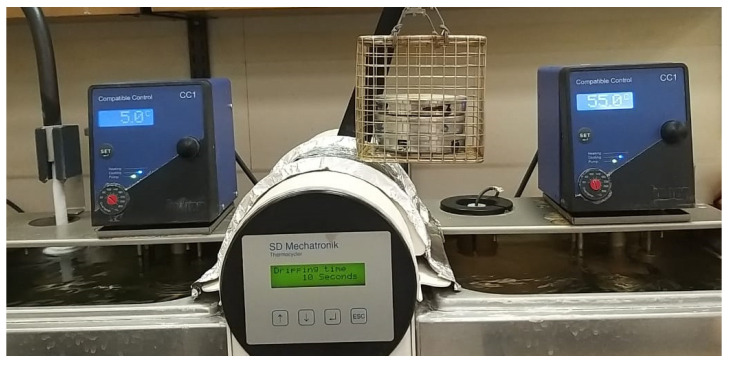
The specimens in the thermocycling unit.

**Figure 8:**
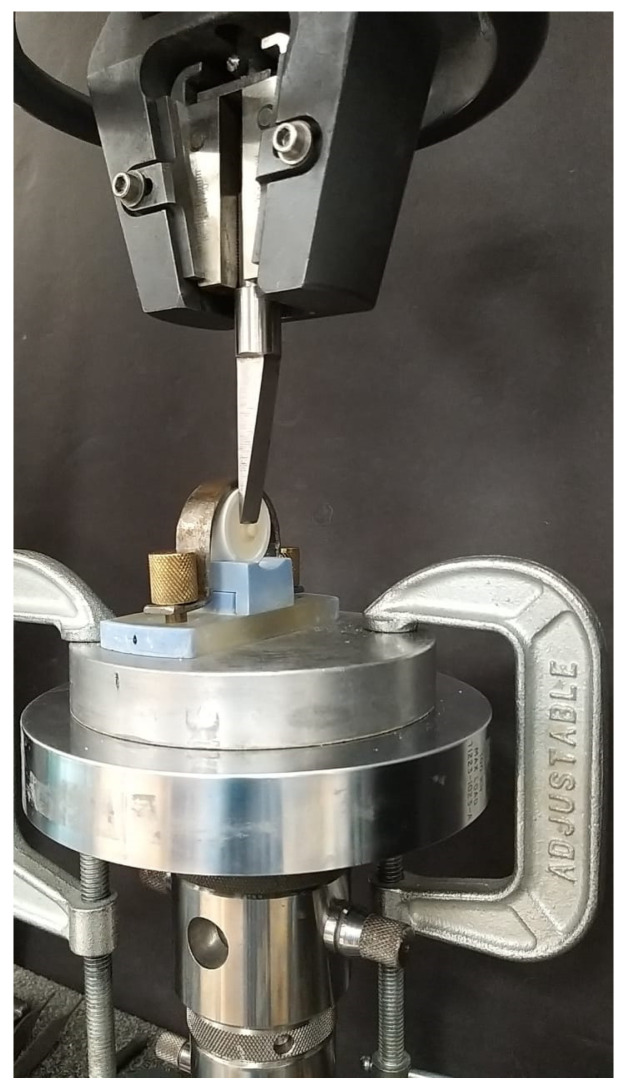
Shear bond strength testing.

**Figure 9:**
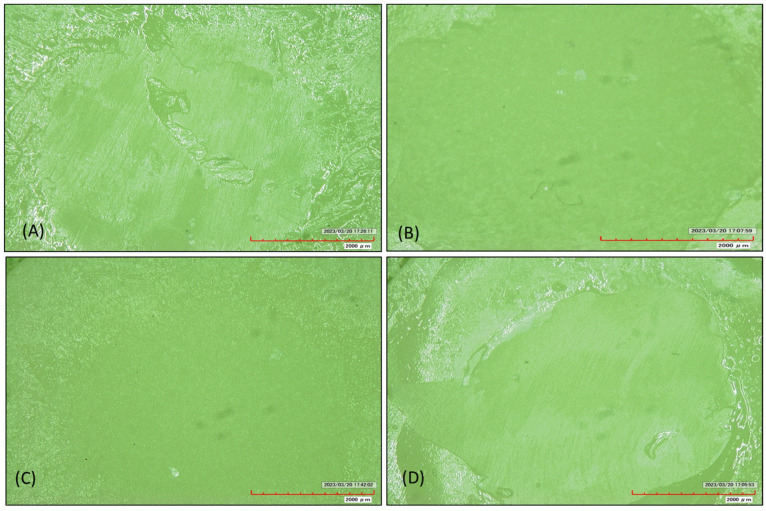
Examples of the samples from different groups, which were used to analyze the fracture patterns with a scanning electron microscope. (**A**) A milled specimen repaired by PMMA, thereby displaying a mixed cohesive and adhesive fracture. (**B**) A milled sample repaired by a composite, thereby displaying a cohesive fracture. (**C**) A 3D-printed sample repaired by PMMA, thereby showing an adhesive fracture. (**D**) A 3D-printed sample repaired by a composite, thereby displaying a mixed cohesive and adhesive fracture.

The base cylinders along with the repair cylinders were subjected to thermocycling in a thermocycling unit (SD Mechatronik, Bayern, Germany) from 5 °C to 55 °C for up to 5000 thermal cycles (dwell time of 30 s), thereby simulating a 6-month period of clinical service (Figure 7). An Instron 3345 (Instron, Norwood, MA, USA) was used to mount the specimens with a customized stainless-steel holder and a jig; this was conducted at a crosshead speed of 0.5 mm/min (Figure 8). A crosshead speed of 0.5 mm/min was used to apply the load directly at the interface between the base cylinder and the repair cylinder until fracture. The shear bond strength (MPa) was calculated by dividing the fracture load (N) by the area of the bonded interface (mm^2^). After the test, each fractured surface was examined by field emission scanning electron microscopy (FE-SEM S4700, Hitachi, Tokyo, Japan), for the fractography analysis. Each sample’s failure mode (cohesive, adhesive, or mixed) was identified by a blinded examiner using a stereomicroscope at a magnification of 40 (Figure 9). Failure at contact between the flowable composite and the temporary crown and bridge material was where the adhesive failure was noticed. When just the flowable composite or the temporary crown and bridge material failed, this was known as cohesive failure. An adhesive failure with a cohesive failure of either of the nearby substrates (flowable composite/PMMA/temporary crown and bridge material) was known as a mixed failure.

### 2.5. Statistical Analysis

The IBM statistical software for the social sciences (SPSS, version 25, IBM Corporation, New York, NY, USA), was used to analyze the data. Three-way ANOVA was used to analyze the effects of each independent component (the material and the bonding condition), as well as the interaction between the independent factors on shear bond strength. Tukey multiple post-hoc tests were used to compare the mean results for each material under various bonding circumstances. The cut off for significance was 5%.

## 3. Results

Based on microscopic examination (Figure 9), most of the milled samples repaired by PMMA (90%) and a composite (87%) showed mixed failure at the fractured surface, while the others in those groups showed cohesive failure. Furthermore, 100% of the 3D-printed samples repaired by a composite showed mixed damage, while 90% of the 3D-printed samples repaired by PMMA showed a mixed fracture and 10% displayed adhesion damage at the cracked surface (Table 4).

The primary groups (milled and 3D-printed) showed significant differences in the shear bond strength (F = 270.9076, *p* = 0.0001). The 3D-printed samples had an increased mean SBS when compared to the milled samples. The composite material repaired group had a greater mean SBS than the PMMA repaired group (F = 259.2048, *p* = 0.0001). The SBS differed significantly among the four milled group subgroups (MC, MCA, MCE, and MCC) (F = 24.0838, *p* = 0.0001) (Table 5). Groups MCA and MCC had a significantly greater mean SBS than Groups MC and MCE. The primary groups (milled and 3D-printed) and materials (composite material and PMMA repair) also showed significant differences in the SBS (F = 65.1731, *p* = 0.0001). The 3D-printed group with composite material had the highest mean SBS, while the milled group with PMMA repair had the lowest. Other groups fell in between. The interactions between the main groups (milled and 3D-printed) and the four subgroups (Group MC, Group MCA, Group MCE, and Group MCC) also affected the SBS (F = 44.2177, *p* = 0.0001). The 3D-printed group with Group MC and Group MCE had the highest mean SBS, while the milled group had the lowest. Other groups had intermediate findings. Composite material and PMMA repair interactions with the four subgroups (Group MC, Group MCA, Group MCE, and Group MCC) significantly affected the SBS (F = 43.4077, *p* = 0.0001). Composite material with Group MCA and Group MCC had the highest mean SBS, whereas PMMA repair with Group MC and Group MCC had the lowest. Other groups had various strengths. Finally, the SBS was affected by the main groups, materials, and four subgroups (F = 23.0496, *p* = 0.0001). The mean SBS varied between the primary groups, materials, and subgroups.

In terms of the SBS, there were significant differences between Group MC and the Groups MCA, MCE, and MCC (*p* < 0.05). This shows that Group MC had a much lower mean SBS than Groups MCA, MCE, and MCC. Additionally, a substantial difference between Group MCA and Group MCE was discovered, with the mean SBS in Group MCE being significantly lower than in Group MCA. However, there was no discernible difference between Group MCA and Group MCC, thereby suggesting that the mean SBS in these two groups was comparable. The lack of a significant difference between Group MCE and Group MCC also suggested that the mean SBS in these two groups was comparable. These results demonstrate the differences in the SBS between the various subgroups, as well as shed light on how the surface conditioning methods and repair materials affect the overall durability of the restored temporary restorations (Table 6).

When comparing the 3D-printed group with the composite material to the milled group with composite material, as well as the milled group with PMMA repair, significant variations in the SBS were found (*p* < 0.05). This shows that, when compared to the milled group with PMMA repair and the 3D-printed group with composite material, the mean SBS in the group with composite material was much lower. In terms of the SBS, there were also significant differences between the milled group with PMMA repair, the 3D-printed group with composite material, and the 3D-printed group with PMMA repair (*p* < 0.05). In comparison to both the 3D-printed group with composite material and the 3D-printed group with PMMA repair, the mean SBS was considerably lower in the milled group with PMMA repair. In terms of the SBS, there was a significant difference between the 3D-printed groups using composite material and the 3D-printed groups using PMMA repair (*p* < 0.05). When compared to the 3D-printed group using composite material, the mean SBS in the PMMA repair group was noticeably lower. These results demonstrate the differences in the SBS between the various combinations of the main groups (milled group and 3D-printed group) and materials (composite material and PMMA repair), and they offer important new information about the effects of the fabrication method and repair material used on the overall strength of the repaired provisional restorations (Table 7).

The results of the statistical analysis, which was carried out at a 5% level of significance, showed a notable difference in the mean SBS between the different pairs. When compared to the milled group with Group MC and Group MCE, the 3D-printed group with Group MC and Group MCE showed a much greater mean SBS. Additionally, when compared to the other groups, the milled group with Groups MC and MCE had the lowest mean SBS. These results indicate that when paired with Group MC and Group MCE, the 3D-printed group demonstrated a greater SBS, but the milled group showed a somewhat lower SBS (Table 8).

At a 5% level of significance, the examination of the data in the table above indicated substantial variations in the mean SBS between certain pairs. Notably, the composite material mixed with Group MCA and Group MCC was shown to have a significantly greater mean SBS. On the other hand, when the PMMA repair was combined with Group MC and Group MCC, the mean SBS was shown to be the lowest. These results underline the differences in the SBS between the various material and subgroup combinations, thus highlighting the superior performance of the composite material when combined with Groups MCA and MCC, as well as the comparably lower SBS observed with PMMA repair when Groups MC and MCC were present (Table 9).

**Figure 10:**
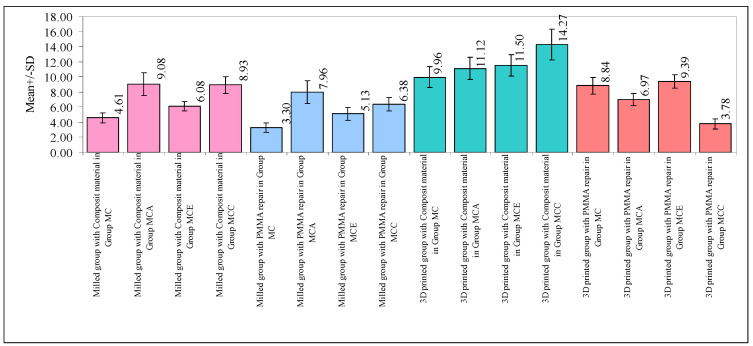
Comparison of the interactions of the two main groups, two materials, and four subgroups with the SBS.

A comparison of the interactions of the two main groups, two materials, and four subgroups with the SBS are presented in Figure 10.

**Figure 11:**
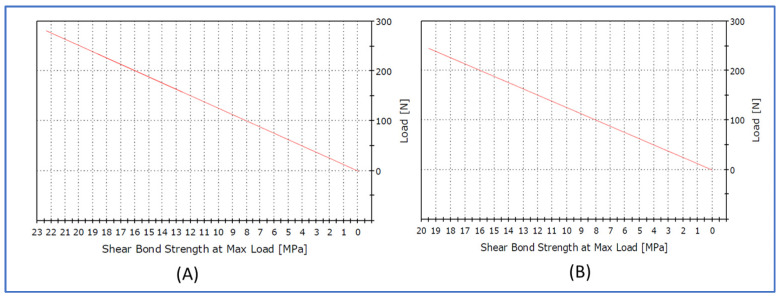
Graph showing the bond strength of the positive controls in relation to maximum load applied. (**A**) Milled positive control. (**B**) 3D-printed positive control.

Figure 11 shows the bond strength of the positive controls in relation to the maximum load applied. The bond strength of the 3D-milled positive control was 22.34 MPa and that of the 3D-printed positive control was 19.49 MPa, which was much higher than the bond strength of the repaired specimens (Figure 10).

## 4. Discussion

The findings of the SBS for the repaired provisional materials that were 3D-printed and milled using various resin materials, with and without chemical and mechanical surface treatment, offer important insights into the functionality and efficacy of these procedures. The objective of this comparative discussion is to evaluate and interpret the results in light of the body of previous studies, thereby emphasizing the implications and potential therapeutic significance of the results that were observed.

First, a statistically significant difference between the fabrication methods (milled groups and 3D-printed groups) was discovered to have an impact on the SBS [21]. When compared to the milled group, the 3D-printed group’s mean SBS was noticeably higher. This result is consistent with earlier research that found that 3D-printed materials had enhanced mechanical properties and bonding strength as a result of their improved interlayer adhesion [22,23,24,25,26,27]. Layering is used to create three-dimensionally printable materials, and, as a result, there is a chemical link between the layers [23]. The mechanical characteristics of the 3D-printed resins were influenced by the fabrication method. The orientation during printing had an impact on the mechanical characteristics [23]. The mechanical characteristics of these materials were also influenced by the layer thickness used during printing [24]. The 3D-printed materials also underwent post-curing after manufacturing, which boosted the degree of conversion and resulted in less residual monomers and improved mechanical characteristics [25]. The increased bonding interfaces produced by the layer-by-layer fabrication and material features inherent to the printing process may be the cause of the better SBS that was observed in the 3D-printed group.

Additionally, it was discovered that the primary impact of the repair materials (composite material versus PMMA repair) on the SBS was statistically significant [21]. When comparing the composite material group to the PMMA repair group, the mean SBS was noticeably higher in the composite material group. This result was in line with earlier research that showed composite materials to have superior mechanical and adhesive capabilities [19,20]. Due to their resin matrix composition and the use of fillers, composite materials have a number of benefits, including greater aesthetics, better marginal adaptability, and higher bond strength [28]. In contrast, PMMA repair materials might have weaker bonds because of their natural traits and reduced bonding capacity [29].

A statistically significant interaction between the fabrication technique and the repair materials on the SBS was discovered [2]. In particular, the composite material and 3D-printed group displayed the highest mean SBS, whereas the milling group and PMMA repair group displayed the lowest mean SBS. These results imply that the best circumstances for attaining the best bonding strength in restored provisional restorations are those that result from the use of 3D printing and composite materials. This can be due to the beneficial interactions between the composite materials’ greater adhesive qualities and the improved bonding surfaces in 3D-printed materials.

Additionally, it was discovered that the interactions between the repair materials, subgroups, and the fabrication process were statistically significant [2]. These findings suggest that the combination of the repair materials and the surface conditioning processes (air-abrasion, etch-primer, and combination) can have a considerable impact on the SBS of the restored temporary prosthesis. These results are in line with other research that has emphasized the significance of surface conditioning and surface treatment methods in strengthening bond strength and in improving the adhesive characteristics of repaired restorations [12,16]. Improved surface roughness and micromechanical retention are produced by surface conditioning treatments including air abrasion and the application of an etch primer, which facilitate the improved adhesion of the repair materials to the temporary prosthesis [14].

Most of the milled samples repaired by PMMA (90%), a composite (87%), 3D-printed samples repaired by PMMA (90%), and all of the 3D-printed samples repaired by a composite (100%) showed a mixed failure at the fractured surface. This indicated that repairs with composite resin and PMMA for milled and 3D-printed samples provided a stable bonding performance.

It is important to note that the observed variations in the SBS among the different subgroups, combinations of fabrication method, and repair materials had significant therapeutic consequences. According to the research, the durability and duration of the repaired provisional restorations can be considerably impacted by choosing the right manufacturing method, repair material, and/or surface conditioning treatments. The clinical effectiveness and success rates of these restorations may be improved by using 3D-printing technology in conjunction with composite materials and the proper surface conditioning procedures.

It is crucial to recognize the limitations of this study. The study’s in vitro setting might not accurately reflect the intricacies and changing variables observed in the oral environment. Furthermore, because different elements including material composition, application procedures, and patient-specific variables can affect the bonding outcomes, the specific resin materials and surface conditioning techniques used in this study might not be generalizable to all clinical scenarios.

## 5. Conclusions

The findings of this study demonstrate the importance of surface conditioning processes, repair materials, and manufacturing methods on the shear bond strength of repaired 3D-printed and milled provisional materials. The results underline the significance of using the right surface conditioning techniques to improve the adhesive characteristics of corrected provisional restorations, as well as support the benefits of 3D-printing technology and composite materials in obtaining improved bonding strength. To validate the results of this study and improve the selection and use of these materials and techniques in clinical practice, future research should look into the long-term clinical performance, as well as assess the bond strength under various loading circumstances.

## Figures and Tables

**Table 1:** Test materials, composition, and manufacturer detail.

Machines/Materials	Manufacturer	Composition	Lot No./Model No.
Five-axis milling machine	DG SHAPE, Roland DGA, Irvine, CA, USA		DWX-52D
3D-printing material	Asiga Pvt Ltd., Alexandria, Australia	7,7,9(or 7,9,9)-trimethyl-4,13-dioxo-3,14-dioxa-5,12-diazahexadecane-1,16-diyl bismethacrylate, tetrahydrofurfuryl methacrylate, diphenyl(2,4,6-trimethylbenzoyl) phosphine oxide	MO/16020
3D-printing machine	Asiga 3D printer, Alexandria, Australia		PN01233
Copra temp	WhitePeaks Dental Solutions GmbH, Wesel, Germany	PMMA (polymethylmethacrylate)/pigments	P10690
Korox 250	BEGO East Coast, New England, Northeastern USA	Aluminum oxide particles 250 µm	1825064
Thermocycling unit	SD Mechatronik, Bayern, Germany		1100
Instron	Instron, Norwood, MA, USA		3345
Field emission Scanning electron microscope	Hitachi, Tokyo, Japan		FE-SEM S4700
Bonding agent			
Meta P & Bond Condac Porcelana Silane Tetric N-Bond	METABIOMED Co., Ltd., Cheongwon-Gun Chungcheongbuk-Do, South Korea FGM dental Products, Brazil Ultradent Products Inc., South Jordan, UT, USA Ivoclar Vivadent, Schaan, Liechtenstein	Bisphenol A glycerolate dimethacrylate, urethane dimethacrylate, pyromellitic glyceryl dimethacrylate, 2-hydroxy ethyl methacrylate, ethyl alcohol 10% hydrofluoric acid 2–1.2 mm ultradent silane hydrofluoric acid (<10%) phosphoric acid acrylate, HEMA, Bis-GMA, urethane dimethacrylate, ethanol, film-forming agent, initiators, and stabilizers	MET2110221 240322 BLG9B Z01PMJ
Repair materials Tetric N-Flow Refill UNIFAST III	Ivoclar Vivadent, Schaan, Liechtenstein GC Asia Dental Pte Ltd., Singapore	36 wt.% dimethacrylate (TEGDMA) 63 wt.% fillers (barium glass, ytterbium trifluoride, highly dispersed silica and mixed oxide). 1 wt.% initiators, stabilizers and pigments, PMMA, dimethyl-p-toluidine benzyl peroxide	Z01W37 2112081

**Table 2:** Grouping for milled base cylinders.

Group	Number of Specimens	Base Cylinder Material	Surface Treatments	Repair Material
Group MC: Control	10	Milled	No treatment	Composite
Group MCA	10	Milled	Air abrasion	Composite
Group MCE	10	Milled	Etch-primer	Composite
Group MCC	10	Milled	Combination (air abrasion and etch-primer)	Composite
Group MP: Control	10	Milled	No treatment	PMMA
Group MPA	10	Milled	Air abrasion	PMMA
Group MPE	10	Milled	Etch-primer	PMMA
Group MPC	10	Milled	Combination (air abrasion and etch-primer)	PMMA

**Table 3:** Groupings for the different 3D-printed base cylinders.

Group	Number of Specimens	Base Cylinder Material	Surface Treatments	Repair Material
Group PC: Control	10	3D-printed	No treatment	Composite
Group PCA	10	3D-printed	Air abrasion	Composite
Group PCE	10	3D-printed	Etch-primer	Composite
Group PCC	10	3D-printed	Combination (air abrasion and etch-primer)	Composite
Group PP: Control	10	3D-printed	No treatment	PMMA
Group PPA	10	3D-printed	Air abrasion	PMMA
Group PPE	10	3D-printed	Etch-primer	PMMA
Group PPC	10	3D-printed	Combination (air abrasion and etch-primer)	PMMA

**Table 4:** Qualitative analyses of the fracture patterns, as seen under the scanning electron microscope.

Type of Specimen	Cohesive	Adhesive	Mixed
Milled specimen repaired by PMMA	10% (4 samples)	-	90% (36 samples)
Milled sample repaired by composite	13% (5 samples)	-	87% (35 samples)
3D-printed sample repaired by PMMA	-	10% (4 samples)	90% (36 samples)
3D-printed sample repaired by composite	-	-	100% (40 samples)

**Table 5:** Comparison of the two main groups, the two materials, and the four subgroups with the SBS by three-way ANOVA.

Sources of Variation	Degrees of Freedom	Sum of Squares	Mean Sum of Squares	F-Value	*p*-Value
Main effects					
Main groups	1	370.89	370.89	270.9076	0.0001 *
Materials	1	354.86	354.86	259.2048	0.0001 *
Subgroups	3	98.92	32.97	24.0838	0.0001 *
2-way interaction effects					
Main groups × Materials	1	89.23	89.23	65.1731	0.0001 *
Main groups × Subgroups	3	181.61	60.54	44.2177	0.0001 *
Materials × Subgroups	3	178.28	59.43	43.4077	0.0001 *
3-way interaction effects					
Main groups × Materials × Subgroups	3	94.67	31.56	23.0496	0.0001 *
Error	144	197.14	1.37	-	-
Total	159	1565.60	-	-	-

* *p* < 0.05.

**Table 6:** Comparison of four subgroups with the SBS by Tukey multiple post-hoc tests.

Subgroups	Group MC	Group MCA	Group MCE	Group MCC
Mean	6.68	8.78	8.02	8.34
SD	2.98	2.03	2.76	4.12
	*p*-value			
Group MC	-			
Group MCA	0.0001 *	-	-	-
Group MCE	0.0001 *	0.0196 *	-	-
Group MCC	0.0001 *	0.3292	0.6205	-

* *p* < 0.05.

**Table 7:** Comparison of the interactions of the two groups and two materials with the SBS via Tukey multiple post-hoc procedures.

Main Groups × Materials	Milled Group with Composite Material	Milled Group with PMMA Repair	3D-Printed Group with Composite Material	3D-Printed Group with PMMA Repair
Mean	7.18	5.69	11.72	7.24
SD	2.17	1.99	2.22	2.38
	*p*-value			
Milled group with composite material	-	-	-	-
Milled group with PMMA repair	0.0001 *	-	-	-
3D-printed group with composite material	0.0001 *	0.0001 *	-	-
3D-printed group with PMMA repair	0.9942	0.0001 *	0.0001 *	-

* *p* < 0.05.

**Table 8:** Comparison of the interactions of the two groups and four subgroups with the SBS via Tukey multiple post-hoc tests.

Main Groups × Subgroups	Milled Group with Group MC	Milled Group with Group MCA	Milled Group with Group MCE	Milled Group with Group MCC	3D-Printed Group with Group MC	3D-Printed Group with Group MCA	3D-Printed Group with Group MCE	3D-Printed Group with Group MCC
Mean	3.95	8.52	5.60	7.66	9.40	9.05	10.45	9.03
SD	0.92	1.58	0.87	1.63	1.34	2.42	1.59	5.59
	*p*-value							
Milled group with Group MC	-	-	-	-	-	-	-	-
Milled group with Group MCA	0.0001 *	-	-	-	-	-	-	-
Milled group with Group MCE	0.0002	0.0001 *	-	-	-	-	-	-
Milled group with Group MCC	0.0001 *	0.2731	0.0001 *	-	-	-	-	-
3D-printed group with Group MC	0.0001 *	*p* = 0.2533	0.0001 *	0.0001 *	-	-	-	-
3D-printed group with Group MCA	0.0001 *	0.8509	0.0001 *	0.0044 *	0.9797 *	-	-	-
3D-printed group with Group MCE	0.0001 *	0.0001 *	0.0001 *	0.0001 *	0.0894	0.0039 *	-	-
3D-printed group with Group MCC	0.0001 *	0.8741	0.0001 *	0.0053 *	0.9727	1.0000	0.0032 *	-

* *p* < 0.05.

**Table 9:** Comparison of the interactions of the two materials and four subgroups with the SBS via Tukey multiple post-hoc procedures.

Main Groups × Subgroups	Composite Material with Group MC	Composite Material with Group MCA	Composite Material with Group MCE	Composite Material with Group MCC	PMMA Repair with Group MC	PMMA Repair with Group MCA	PMMA Repair with Group MCE	PMMA Repair with Group MCC
Mean	7.29	10.10	8.79	11.60	6.07	7.46	7.26	5.08
SD	2.94	1.78	2.98	3.18	2.97	1.29	2.35	1.54
	*p*-value							
Composite material with Group MC	-	--	-	-	-	-	-	-
Composite material with Group MCA	0.0001 *	-	-	-	-	-	-	-
Composite material with Group MCE	0.0012 *	0.0097 *	-	-	-	-	-	-
Composite material with Group MCC	0.0001 *	0.0013 *	0.0001 *	-	-	-	-	-
PMMA repair with Group MC	0.0223	0.0001 *	0.0001 *	0.0001 *	-	-	-	-
PMMA repair with Group MCA	0.9997	0.0001 *	0.0078	0.0001 *	0.0040 *	-	-	-
PMMA repair with Group MCE	0.0000	0.0001 *	0.0009 *	0.0001 *	0.0291 *	0.9993	-	-
PMMA repair with Group MCC	0.0001 *	0.0001 *	0.0001 *	0.0001 *	0.1328	0.0001 *	0.0001 *	-

* *p* < 0.05.

## Data Availability

The data that support the findings of this study are available from the corresponding authors upon reasonable request.
